# Characterization of an Amphiphilic Phosphonated Calixarene Carrier Loaded With Carboplatin and Paclitaxel: A Preliminary Study to Treat Colon Cancer *in vitro* and *in vivo*

**DOI:** 10.3389/fbioe.2019.00238

**Published:** 2019-10-01

**Authors:** Meiying Li, Liujun Mao, Meirong Chen, Mingxin Li, Kaixuan Wang, Jingxin Mo

**Affiliations:** ^1^Clinical Research Center for Neurological Diseases of Guangxi Province, Affiliated Hospital of Guilin Medical University, Guilin, China; ^2^School of Pharmacy, Guilin Medical University, Guilin, China; ^3^Department of Further-Education, Affiliated Hospital of Guilin Medical University, Guilin, China; ^4^Department of Graduate, Affiliated Hospital of Guilin Medical University, Guilin, China

**Keywords:** phosphonated calixarene, paclitaxel, carboplatin, nanomedicine, colon cancer

## Abstract

The inadequacy of available detection methods and a naturally aggressive progression have made colon cancer the third most common type of cancer, accounting for ~10% of all cancer cases. The heterogeneity and genomic instability of colon cancer tumors make current treatments unsatisfactory. This study evaluated a novel nanoscale delivery platform comprising phosphonated calixarenes (P4C6) co-loaded with paclitaxel (PTX) and carboplatin (CPT). The nanoparticles showed average hydrodynamic sizes of 84 ± 8 nm for empty P4C6 nanoparticle and 119 ± 13 nm for PTX-CPT-P4C6. The corresponding zeta potentials were −40.8 ± 8.8 and −35.4 ± 4.2 mV. The optimal CPT:PTX ratio was 5.22:1, and PTX-CPT-P4C6 with this ratio was more cytotoxic against HT-29 cells than against Caco-2 cells (IC_50_, 0.4 ± 0.02 vs. 2.1 ± 0.3 μM), and it induced higher apoptosis in HT-29 cells (56.6 ± 4.5 vs. 44.9 ± 3.44%). PTX-CPT-P4C6 inhibited the invasion and migration of HT-29 cells more strongly than the free drugs. It also inhibited the growth of HT-29 tumors in mice to the greatest extent of all formulations, with negligible side effects. This research demonstrates the potential of P4C6 to deliver two chemotherapeutic agents to colon cancer tumors to provide synergistic efficacy than single drug administration.

## Introduction

Colorectal cancer (colon cancer) is the third most common type of cancer worldwide and a leading cause of cancer death, making up about 10% of all cancer cases (den Bakker et al., 2018). In 2012, its global mortality rate was estimated at more than 600,000 per year, and its incidence has increased over the last 25 years (Yodkeeree et al., 2009). Colon cancer manifests as the formation of adenomatous polyps and malignant cells in the colon (Basu et al., 2018). Its naturally aggressive progression, in combination with a lack of accurate screening and detection methods, means that patients are typically diagnosed at advanced stages, and they therefore respond inadequately to available treatments (Blind et al., 2018). Chemotherapies are essential due to the high risk of relapse after surgery, yet most drugs for treating advanced-stage colorectal cancer, such as cisplatin, are relatively ineffective and frequently induce adverse side effects (Rabik and Dolan, 2007; Robella et al., 2019), such as nephro-(Zhu et al., 2019), hepato-(Zhang X. et al., 2019), and cardiotoxicity (Dasari and Tchounwou, 2014). These considerations highlight the need for new therapeutic approaches for this disease.

Nanomedicine is an emerging, dynamic branch of therapeutics that continues to gain prominence as a viable treatment alternative for many cancers, including colon cancer (Zhang Q. et al., 2019). Nanomedicine encompasses the application of nanotechnology (construction of functional structures on the nanometer scale) to the treatment, diagnosis, monitoring, and control of biological systems (Yang et al., 2015; Li et al., 2019; Wei et al., 2019). This field has seen the development of a number of drug delivery platforms, including polymer-drug conjugates (Li and Wallace, 2008; Karolczak-Bayatti et al., 2019), liposomes (Paasonen et al., 2010; Shen and Ye, 2019), micelles (Dehghan Kelishady et al., 2014; Alliot et al., 2019), nanoshells (Huschka et al., 2012; Russo et al., 2019), and dendrimers (Modi et al., 2014; Zhao et al., 2017). The overarching aim of nanomedicine development is to design more specific drug delivery and targeting therapies as alternatives to conventional therapies.

Paclitaxel (PTX) and carboplatin (CPT) are first-line cancer chemotherapy (Barcelos et al., 2019). PTX (MW 853.9 g/mol) is a hydrophobic molecule that suppresses dynamic instability of microtubules and thereby inhibits mitosis (Nogales and Wang, 2006). CPT (MW 371.3 g/mol) is a second-generation analog of the platinum complex called carboplatin (Zhang et al., 2016). CPT reacts with genomic DNA to yield a variety of cross-linked adducts within and between DNA strands as well as between DNA and proteins, which interfere with DNA transcription (Rabik and Dolan, 2007; Thibault et al., 2018). When used together, PTX and CPT can show effective synergistic anti-cancer activity, yet they also frequently cause toxicity that reduces quality of life (Tourell et al., 2017). Toxicities associated with CPT involve mainly myelosuppression, principally thrombocytopenia (Nunes et al., 2018). PTX toxicity arises not only from the drug itself but also from the high concentration of the Cremophor EL vehicle, which is required to solubilize the poorly aqueous PTX into an injectable solution. The most common toxicities associated with Cremophor EL include acute hypersensitivity reactions, which are recognizable as flushing, rash, dyspnea and tachycardia (Bhatt et al., 2019; Bressand et al., 2019).

**Figure 1:**
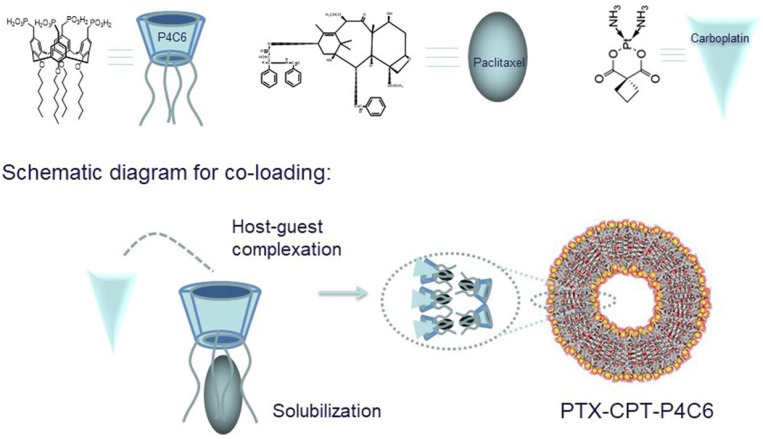
Calixarene cone formation creates a bowl-shaped cavity for CPT and an internal core for PTX, giving rise to a dual-loaded nanoparticle (PTX-CPT-P4C6).

Here we developed a novel nanoscale delivery platform that allows co-delivery of PTX and CPT in a formulation that reduces the drugs' toxic effects and potentially improves efficacy. The platform comprises amphiphilic phosphonated calixarene (P4C6) assembled into a supramolecular cone. The P4C6 molecule consists of calix[4]arene with four ionizable phosphonic acid groups attached to the upper rim and four six-carbon alkyl moieties attached to the lower rim (Figure 1). The alkylated analogs of phosphonated calix[4]arenes are amphiphilic molecules that can self-assemble via interactions between the polar head groups and hydrophobic interactions between the alkyl chains (Mo et al., 2015, 2016, 2017; Chen et al., 2017). In this way, P4C6 molecules self-assemble into a liposomal structure, with the hydrophilic ${\text{PO}}_{3}^{-3}$ groups extending into the aqueous environment and the internal hydrophobic alkyl chains forming the layers of liposome. Through a combination of liposomal and host-guest drug-loading techniques, our laboratory has succeeded in loading hydrophobic PTX into the core of P4C6 nanoparticles and hydrophilic CPT into the anionic “bowl” of individual P4C6 molecules (Mo et al., 2015, 2016, 2017).

In the present study, we characterized the size of empty and drug-loaded P4C6 carriers over a range of biologically relevant pH values, determined the optimized ratio between PTX and CPT, and evaluated nanoparticle cytotoxicity against colon cancer cell lines. Finally, we evaluated the ability of the nanoparticles to inhibit colon cancer tumor growth in mice.

## Materials and Methods

### Materials

All materials and reagents were purchased from commercial sources and used as received. CPT, dimethyl sulfoxide (DMSO), 3-[4,5-dimethylthiazol-2-yl]-2,5-diphenyltetrazolium bromide (MTT) and trypan blue were purchased from Sigma-Aldrich (St. Louis, MO, USA). Phosphate-buffered saline (PBS), fetal bovine serum (FBS), penicillin/streptomycin, trypLE express enzyme, McCoy's 5A media and Ham's F-12K (Kaighn's) nutrient mix were purchased from Life Technologies (Carlsbad, CA, USA). Paclitaxel was sourced from 21 CEC PX Pharm Ltd., (East Sussex, UK); chloroform and hydrochloric acid, from APS Chemicals (Canning Vale, WA, Australia); and sodium hydroxide, from Ajax FineChem (Scoresby, VIC, Australia). P4C6 was synthesized in our lab to a purity of >95%, as confirmed by HPLC (Mo et al., 2015).

### Cell Culture

The two human colon cancer cell lines Caco-2, which show features of colonic epithelial cells, and HT-29, which resemble colonic crypt cells, were obtained from the American Type Culture Collection (ATCC). Caco-2 cells were grown in McCoy's 5A medium supplemented with FBS (10% v/v) and penicillin-streptomycin (1% v/v). HT-29 cells were grown in Ham's F-12K (Kaighn's) medium supplemented with FBS (10% v/v) and penicillin-streptomycin (1% v/v). When cells reached 80–100% confluence, they were trypsinized with trypLE express enzyme, centrifuged at 300 g for 3 min in a 2-16PK refrigerated centrifuge (Sigma Laborzentrifugen, Osterode am Harz, Germany), and split 1:4 in fresh medium.

### Synthesis of Compound P4C6

P4C6 was synthesized as described previously (Mo et al., 2015). Briefly, n-hexyl groups were attached to the lower rim of calix[4]arene reaction with bromohexane and sodium hydride in DMF, the so-called Duff reaction enabled formylation, and the formylated compound was reduced to alcohol on the upper rime of calix[4]arene by sodium borohydride. The alcoholic group was chlorinated by thionyl chloride, phosphorylated by triethylphosphite and finally deprotected by bromotrimethylsilane. The chemical structure of the resultant P4C6 was confirmed by ^1^H NMR (Mercury 400, Varian, Palo Alto, CA; Figure S1).

### Preparation of PTX-CPT Mixture

PTX and CPT were precisely weighed and dissolved, respectively, in 1% DMSO or pure water to a final concentration of 1 mg/ml. These stock solutions were mixed to obtain different ratios of PTX:CPT for subsequent experiments.

### Preparation of PTX- and/or CPT-Loaded P4C6 Nanoparticles and Empty P4C6 Nanoparticles

PTX- and/or CPT-loaded P4C6 nanoparticles were prepared as we described previously (Mo et al., 2017). Briefly, P4C6 (150 mg) was mixed with PTX (5 mg) in 50 mL of ethyl acetate in a 150-mL round-bottom flask. The flask was left on a rotary evaporator (Buchi) overnight in a 40°C water bath to eliminate ethyl acetate. The resulting thin film was rehydrated at 40°C for 30 min in 30 mL of deionized water containing CPT (10 mg), after which the suspension was sonicated for 10 min using a probe sonicator (Qsonica L.L.C, Newtown, CT, USA; 500 W, 220 V) at 50% strength. The solution was passed through a 0.5-μm filter (Millipore) to remove insoluble material, and the resulting PTX-CPT-P4C6 nanoparticles were freeze-dried and stored at −20°C until experiments. PTX- or CPT-loaded P4C6 nanoparticles or empty P4C6 nanoparticles were prepared following the same procedure as above but leaving out PTX and/or CPT.

### Physicochemical Characterization of Nanoparticles

Each batch of nanoparticles was characterized using dynamic light scattering (DLS) to determine particle size and size distribution, and by electrophoretic light scattering to measure zeta potential. Measurements were made with a 4-mW He-Ne laser at 633 nm and with a measurement angle of 173° (Zetasizer Nano S, Malvern Instruments, Worcestershire, UK). For particle size calculations, we used the refractive index (1.330) and viscosity (0.887) of water at 25°C, and we used its dielectric constant (78.5) for zeta potential measurements. Average values for nanoparticle size and zeta potential were calculated by the equipment, and the polydispersity index was also measured. The concentrations of PTX and CPT were determined using a method based on liquid chromatography and time-of-flight mass spectrometry (LC/TOF MS) as described in Supplementary Information (Mo et al., 2014). The entrapment efficiency (EE) was calculated from the following equation:

$$EE\left(\%\right)\;=\;\frac{amount\;of\;paclitaxle\;\left(or\;carboplatin\right)\;in\;nanoparticle\;pellet\;\left(\mu g\right)}{amount\;of\;paclitaxel\;\left(or\;carboplatin\right)\;in\;nanoparticle\;dispersion\;\left(\mu g\right)}\;\times \;100\%$$

The drug loading (DL) of PTX or CPT in the freeze-dried nanoparticles powder was calculated using the following equation:

$$\begin{array}{l} DL\;\left(\%\right)=\;\frac{amount\;of\;paclitaxel\;\left(or\;carboplatin\right)in\;freeze-dried\;nanoparticle\left(\mu g\right)}{amount\;of\;freeze-dried\;nanoparticle\left(\mu g\right)}\;\times \;100\% \end{array}$$

The EE and DL of PTX and CPT were determined from three separately prepared nanoparticles, and were expressed as the mean ± standard deviation.

For transmission electron microscopy, nanoparticles were diluted 1:30 (v/v) in MilliQ water. One drop of the diluted dispersion was deposited onto copper-coated carbon grids (Polysciences, Inc., Warrington, PA, USA). The grids were dried at ambient temperature for 3 days, then images were collected using a JEM2100 transmission electron microscope (JEOL, Akishim-Shi, Tokyo, Japan) at 120 kV and an 11Mpix Orius digital camera (Gatan, Pleasanton, CA, USA).

### Assessment of Nanoparticle Stability

Particle size and zeta potential measurements were taken at 0, 24, 48, and 72 h after preparation, during incubation in media of pH from 2 to 10. Each dispersion was adjusted to the specified pH by drop-wise addition of HCl to acetate buffer solution (pH 2–6) or NaOH to phosphate buffer solution (pH 7–10), and the pH of the samples was measured using a SevenEasy pH meter (Mettler Toledo, Greifensee, Switzerland). Dilution stability of 0.3 μM PTX-CPT-P4C6 was assessed by dilution with 0.9% saline for up to 100 times. The particle size distribution profiles, polydispersity index (PDI) and zeta potential values of the corresponding samples were immediately characterized by DLS.

### Nanoparticle Cytotoxicity *in vitro*

Caco-2 and HT-29 cells were seeded onto 96-well plates at a concentration of 10,000 cells/well together with 200 μl of McCoy's 5A medium or Ham's F-12K (Kaighn's) medium, respectively. The cells were incubated for 24 h at 5% CO_2_ and 37°C, before the medium in each well was replaced with an equal volume of medium containing the appropriate concentration of samples. Plates were then incubated in a humidified atmosphere containing 5% CO_2_ at 37°C for 2 or 48 h. For experiments involving 2-h exposure, sample-containing medium was aspirated after 2 h, and replaced with 200 μl of fresh, drug-free culture medium. The cells were then incubated another 46 h, and finally analyzed in the MTT assay. For experiments involving 48-h exposure, cells were processed in the MTT assay immediately after the 48-h incubation.

MTT was dissolved in PBS at a concentration of 5 mg/ml. Each well of cells was incubated with 20 μl of this mixture for 3 h, excess MTT was aspirated and DMSO was added (200 μl/well), and plates were incubated for 15 min at ambient temperature away from light. Plates were then analyzed at 595 nm on a Multiskan RC Microplate Reader (Thermo/LabSystems, Champaign, IL, USA). Cell survival percentages relative to control cells exposed to medium without any nanoparticle or drugs were plotted as a function of nanoparticle or drug concentration on a logarithmic scale. A dose-response curve was generated using three-parameter, non-linear inhibition dose-response fitting in Prism 6.05 (GraphPad Software, La Jolla, CA, USA). The half-maximal inhibitory concentration (IC_**50**_) was determined based on this curve.

The chemomodulatory effect of carboplatin (CPT) to paclitaxel (PTX) within colon cancer cells was determined using combination analysis between PTX and CPT as previously described (Chen et al., 2017). Briefly, exponentially growing HT-29 and CaCo-2 cells were seeded in 96-well plates (2,000 cells/well) and exposed to fixed concentrations of PTX and CPT (molar ratio of 5.22:1) for 2 and 48 h. Cells were subsequently subjected to MTT assay as described in the previous section. Combination index (CI-value) was calculated and used to define the nature of drug interaction (synergism if CI-value <0.8 as; antagonism if CI-value >1.2; and additive if CI-value ranges from 0.8 to 1.2). CI value was calculated from the formula:

$$\begin{array}{l} \text{CI value}=\frac{{\text{IC}}_{50}\text{ of drug }\left(\text{PTX}\right)\text{ combination}}{{\text{IC}}_{50}\text{ of drug }\left(\text{PTX}\right)\text{ alone}}\\ \;+\frac{{\text{IC}}_{50}\text{ of drug }\left(\text{CPT}\right)\text{ combination}}{\text{I}{\text{C}}_{50}\text{ of drug }\left(\text{CPT}\right)\text{ alone}} \end{array}$$

### Effects of Nanoparticles on Apoptosis and Cell Cycle Distribution *in vitro*

Caco-2 and HT-29 cells were seeded in 6-well plates with 2 ml of McCoy's 5A medium or Ham's F-12K (Kaighn's) medium, respectively. After allowing cells to adhere for 24 h, the medium was replaced with 2 ml P4C6 (200 μM), PTX-CPT mixture (5 μM PTX and 26.1 μM CPT) or PTX-CPT-P4C6 (0.3 μM PTX and 1.57 μM CPT). Negative controls are Caco-2 and HT-29 cells cultured as the same procedure as above without any treatment. The plates were incubated in a humidified atmosphere containing 5% CO_2_ at 37°C for 48 h (Forma™ Series II Water-Jacketed CO_2_ Incubator, Thermo Fisher Scientific, Waltham, MA, USA).

The cells were then prepared for flow cytometry using a FITC-Annexin V/Dead Cell Apoptosis Kit (Sigma-Aldrich) according to the manufacturer's protocol. Briefly, harvested cells from each well were washed with 1 ml of PBS, centrifuged (300 *g* for 3 min) and the pellet was resuspended in 100 μl 1X annexin-binding buffer, together with 5 μl of FITC and 1 μl of propidium iodide (PI; 100 μg/ml). The sample was incubated for 15 min at ambient temperature, then an additional 400 μl of 1X annexin-binding buffer was added. For cell cycle distribution analysis, the treated and untreated cells were washed and re-suspended in 1 mL of PBS containing 50 μg/mL RNAase A (Sigma-Aldrich) and 10 μg/mL propidium iodide (PI) (Sigma-Aldrich) for 20 min. The samples were kept on ice until analysis. Flow cytometry was performed on a FACS Calibur Flow Cytometer (Becton Dickinson, Franklin Lake, NJ, USA) and the data were analyzed using CellQuest Pro (Becton Dickinson) to determine levels of apoptosis and distribution in the cell cycle.

### Cell Migration and Invasion *in vitro*

*In vitro* cell invasion assays (also known as the Boyden transwell chamber assay) were conducted using 24-well cell culture plates with polycarbonate inserts (Millipore) with pore diameters of 8 μm. In brief, cells (5 × 10^4^) in 300 μL serum-free DMEM were seeded onto the upper chambers for 24 h for cells attached, then 800 μL DMEM with 10 % serum was added to the lower chambers. After incubation for another 24 h with 300 μL serum-free DMEM containing P4C6 (1.73 μg), PTX-CPT mixture (0.077 μg of PTX, 0.174 μg) or PTX-CPT-P4C6 (0.077 μg of PTX, 0.174 μg of CPT, and 1.73 μg of P4C6), the supernatant containing treatments was aspirated. Fresh medium was then added and cells were incubated a further 48 h. Cells that had not penetrated the filter were wiped away with cotton swabs, and cells that had migrated to the lower surface of the filter were counted in five randomly selected fields at 40 × magnification using a light microscope (Olympus, Japan). Each assay was performed in triplicate.

Effects of different formulations on HT-29 cell migratory activity were examined in a wound healing assay. Cells were cultured in six-well plates in a 5% CO_2_ incubator for 24 h until completely confluent. The cell monolayer was scratched with a 200-μL pipette tip to inflict a wound, cells were washed twice in PBS to remove floaters, and the medium was replaced by 2 mL 0.5% FBS DMEM containing P4C6 (11.54 mg) or PTX-CPT mixture (0.51 mg PTX, 1.16 mg CPT) or PTX-CPT-P4C6 (0.51 mg of PTX, 1.16 mg of CPT and 11.54 mg of P4C6). After another 24 h of incubation, the supernatant was aspirated and replaced with fresh medium without any nanoparticles or drugs, and the cultures were incubated another 96 h. Migration of cells from the leading edge was analyzed using light microscopy. Relative gap area was measured using imageJ software (NIH, USA), and the ratio of gap area for each group at 96 h relative to the gap area in each group at 0 h was plotted.

### Anti-tumor Effects of Nanoparticles *in vivo*

Female Balb/c nude mice aged 5–6 weeks were obtained from the Model Animal Research Center of Nanjing University (Nanjing, China). All animal experiments were approved by the Ethics Committee of Guilin Medical University (ethics number YXLL-2016-088). HT-29 cells (2 × 10^6^ in 100 μL PBS) were injected subcutaneously into the upper right thigh of mice. Treatments with different formulations were initiated when tumors had reached a volume of 200 mm^3^. Mice were randomly divided into four groups (5 per group): the empty P4C6 nanoparticles group, 8.73 mg/kg P4C6; the PTX-CPT group, 1.27 mg/kg PTX-CPT (0.39 mg+0.88 mg); the PTX-CPT-P4C6 group, 10 mg/kg PTX-CPT-P4C6 (containing 0.39 mg PTX, 0.88 mg CPT, and 8.73 mg P4C6) and control group, 0.9% saline. These treatments were administered by oral gavage once every other day after body weight and tumor volume had been recorded. Tumor volume was calculated according to the following equation.

$$\begin{array}{l} Tumor\;volume\;\left(mm^{3}\right)=0.5\;\times long\;axis\;\left(mm\right)\\ \;\times {\left[short\;axis\;\left(mm\right)\right]}^{2} \end{array}$$

Tumors were not allowed to grow beyond 3,000 mm^3^, in accordance with our institutional animal care guidelines. Once the maximum tumor volume was reached, the animal was euthanized with pentobarbital.

The mice were euthanized on day 12 of drug administration. Tumor tissues were removed completely and weighed. Tumors, livers, and kidneys were fixed in formalin, embedded in paraffin, thin-sliced, and stained with hematoxylin-eosin for histological analysis. Blood was collected from the postcaval vein, centrifuged at 1,006 *g* for 10 min, and the serum supernatant was assayed for aspartate aminotransferase and blood urea nitrogen levels using a blood autoanalyzer (CDC Technologies, OH, USA).

For staining with Annexin V and PI, the tumors were cut into slices 5.0 μm thick, placed on slides, washed with PBS three times, then incubated with binding buffer containing Annexin V and PI for 15 min at 25°C in the dark. Finally, samples were washed, stained for nuclei using DAPI (5 mM), and examined by confocal microscopy (Leica TCS SP5, Germany).

To further determine the degree of apoptosis-like cell death, terminal deoxynucleotidyl transferase-mediated dUTP end-labeling (TUNEL) (Nanjing Jiancheng Bioengineering Institute, Nanjing, China) was performed. Tumor sections of different treatment groups were processed according to the manufacturer's protocol, and examined using a light microscope (ECLIPSE 80i, Nikon, Tokyo, Japan).

### Statistical Analyses

Data were presented as mean ± SD. Variance analysis and *t*-tests were used to assess the significance of differences. Differences were considered significant at three levels: ^*^p < 0.05, ^**^p < 0.01, and ^***^p < 0.001. All batches were produced in triplicates otherwise mentioned. Each experiment was repeated twice.

## Results

### Physicochemical Characterization of Nanoparticles

In the dual-loaded formulation of PTX-CPT-P4C6, EE and DL were 87.66 ± 6.07 and 3.88 ± 0.86% for PTX, and 56.6 ± 3.88 and 8.79 ± 0.28% for CPT. In the single-loaded formulations of PTX-P4C6 and CPT-P4C6, rather higher EE and DL were observed as 91.28 ± 11.07 and 4.93 ± 0.51% for PTX, and 58.85 ± 5.06 and 11.5 ± 2.8% for CPT (Table 1).

**Table 1:** Characterization of various nanoparticle formulations by DLS and LC/TOF MS.

**Formulations**	**Particle size (nm)**	**PDI**	**Zeta potential (mV)**	**Loading efficiency (%)**
Empty P4C6 nanoparticles	84 ± 8	0.22 ± 0.01	−40.8 ± 8.8	N/A
PTX-CPT-P4C6	119 ± 13	0.21 ± 0.01	−35.4 ± 4.2	3.88 ± 0.86 (PTX) 8.79 ± 0.28 (CPT)
PTX-P4C6	121 ± 13	0.26 ± 0.05	−39.8 ± 9.2	4.93 ± 0.51
CPT-P4C6	130 ± 18	0.22 ± 0.07	−36.6 ± 6.8	11.5 ± 2.8

**Figure 2:**
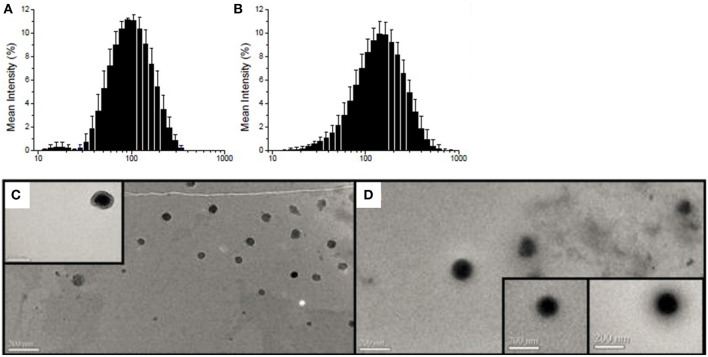
Particle size distribution (nm) as determined by dynamic light scattering: **(A)** empty P4C6 nanoparticles and **(B)** PTX-CPT-P4C6; Transmission electron micrographs of **(C)** empty P4C6 nanoparticles and **(D)** PTX-CPT-P4C6. Scale bar, 200 nm.

Average hydrodynamic diameter and polydispersity index were 84 ± 8 nm and 0.22 ± 0.01 for empty P4C6 nanoparticles, compared to 119 ± 13 nm and 0.21 ± 0.01 for PTX-CPT-P4C6 (Figure 2). Loading P4C6 with both PTX and CPT significantly increased mean particle size (*p* ≤ 0.01). Zeta potential was not significantly different between empty P4C6 nanoparticles (−40.8 ± 8.8 mV) and PTX-CPT-P4C6 (−35.4 ± 4.2 mV). Compared to dual-loaded P4C6 nanoparticles, loading P4C6 with either PTX or CPT would slightly increase the corresponding nanoparticle sizes and loading efficiencies as well. In all nanoparticle formulations, their PDI and zeta potentials were of no comparative differences (Table 1).

Transmission electron microscopy showed empty P4C6 nanoparticles and PTX-CPT-P4C6 nanoparticles to be uniformly round (Figures 2C,D) and to have diameters consistent with those determined by dynamic light scattering.

### Nanoparticle Stability

**Figure 3:**
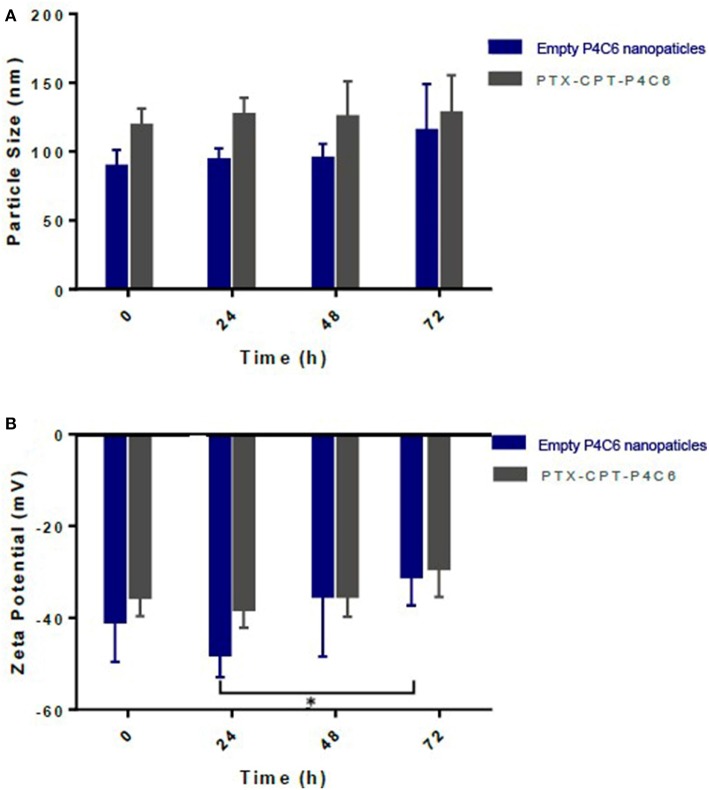
Changes in different nanoparticles during 72-h storage in terms of **(A)** size as measured using dynamic light scattering and **(B)** zeta potential as measured using electrophoretic light scattering.

Figure 3 shows average size of empty P4C6 nanoparticles and PTX-CPT-P4C6 nanoparticles during 72-h storage at ambient temperature in the dark. Neither mean particle size nor zeta potential of PTX-CPT-P4C6 changed significantly during storage (Figure 3). In contrast, empty P4C6 nanoparticles did not change significantly in size, but their zeta potential decreased significantly between 24 and 72 h (*p* ≤ 0.05).

It can be observed from Figure S2 that there were no significant changes in the mean particle size of PTX-CPT-P4C6 upon dilution with 0.9% saline (up to 100 times, *p* > 0.05). At the same time, the polydispersity index and zeta potential slightly increased with multiple times dilution, though the difference is negligible.

**Figure 4:**
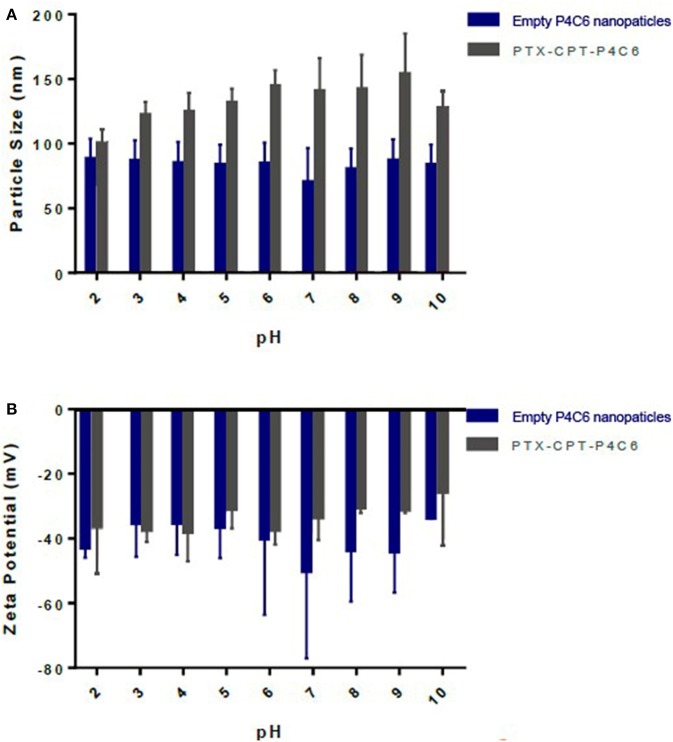
Effects of pH on different nanoparticles **(A)** size and **(B)** zeta potential.

To assess nanoparticle stability across a pharmaceutically relevant pH range, the pH of empty P4C6 nanoparticles and PTX-CPT-P4C6 dispersions was gradually adjusted to specific values (pH 2–10). In no case did mean particle size or size distribution vary substantially at pH values up to 10 (Figure 4A). Similarly, zeta potential did not vary significantly over this pH range (Figure 4B).

### Nanoparticle Cytotoxicity *in vitro*

**Figure 5:**
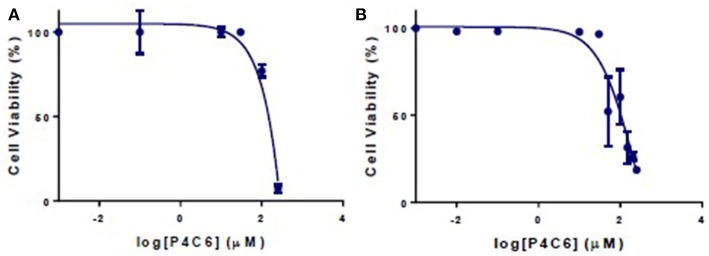
Viability of **(A)** Caco-2 and **(B)** HT-29 cells after 24 h treatment with different concentrations of empty P4C6 nanoparticles. Viability was measured in the MTT assay.

Empty P4C6 nanoparticles showed an IC_**50**_ of 2050.8 ± 2.2 μM against HT-29 cells and 1340.9 ± 1.5 μM against Caco-2 cells (Figure 5).

Next, we determined the IC_50_ of combinations of CPT and PTX mixed in three molar ratios, 5.22:0.5, 5.22:1, and 5.22:2. Our goal was to optimize the ratio of the two drugs in the dual-drug delivery platform PTX-CPT-P4C6. Of the three ratios tested, the ratio 5.22:1 was the most cytotoxic to HT-29 and Caco-2 cells (Figure S3, Table 2), so this ratio was used in all subsequent experiments.

**Table 2:** IC_50_ values of the PTX-CPT mixture prepared in different drug ratios.

**Molar ratio CPT:PTX**	**Against Caco-2 cells**	**Against HT-29 cells**
	**IC_**50**_ (μM)**	**IC_**50**_ (μM)**
5.22:0.5	8.5 ± 0.8	15.9 ± 1.5
5.22:1	7.2 ± 1.4	2.1 ± 0.4
5.22:2	8.2 ± 3.1	3.0 ± 1.3

*Values are mean ± SD in terms of PTX concentration*.

The therapeutic efficacy of this molar ratio was confirmed in two ways. First, PTX-CPT-P4C6 formulated with this ratio showed significantly lower IC_**50**_ at 24 h than the simple PTX-CPT mixture prepared with that ratio (Table 3). Second, dose-response curves showed significantly lower IC_50_ for PTX-CPT-P4C6 than for PTX-CPT mixture at 2 and 48 h (Figure S4, Table 3).

**Table 3:** IC_50_ values (μM) for empty P4C6 nanoparticles, PTX- and/or CPT-P4C6 nanoparticles and the simple PTX and/or CPT mixture when the two drugs were mixed in the molar ratio CPT:PTX = 5.22:1.

	**Caco-2**		**HT-29**	
**Treatments**	**2 h exposure**	**48 h exposure**	**2 h exposure**	**48 h exposure**
	**IC_**50**_ (μM)**	**IC_**50**_ (μM)**	**IC_**50**_ (μM)**	**IC_**50**_ (μM)**
Empty P4C6 nanoparticles	1204.0 ± 94.6	635.1 ± 58.4	806.9 ± 61.5	187.8 ± 14.2
PTX	326.4 ± 28.6	20.8 ± 3.8	117.4 ± 13.4	13.9 ± 2.1
PTX-P4C6	126.2 ± 21	16.4 ± 0.5	79.1 ± 6.4	9.9 ± 1.4
CPT	213.5 ± 18.6	16.2 ± 2.9	140.1 ± 15.9	13.1 ± 2.1
CPT-P4C6	110.2 ± 14.6	12.7 ± 1.6	87.6 ± 6.2	10.9 ± 0.8
PTX-CPT	110.2 ± 21.5	6.2 ± 0.5	62.8 ± 8.4	1.7 ± 0.3
PTX-CPT-P4C6	25.2 ± 3.7	2.1 ± 0.3	4.6 ± 0.3	0.4 ± 0.02
CI-index (CI-value)	Synergism (0.44)	Synergism (0.37)	Synergism (0.62)	Synergism (0.15)

*Data are mean ± SD*.

Under 48 h incubation period, cytotoxicity of CPT was weaker in CaCo-2 cells compared to HT-29 cells with IC_50_s of 16.2 ± 2.9 and 13.1 ± 2.1 μM, respectively. However, CPT synergistically improved the cytotoxic profile of PTX against both colon cancer cell lines. Under 48 h incubation period, CPT significantly decreased the IC_50_s of PTX from 13.9 ± 2.1 and 20.8 ± 3.8 μM to 0.4 ± 0.02 and 2.1 ± 0.3 μM in HT-29 and CaCo-2 cells, respectively. The combination indices for PTX and CPT (molar ratio of 5.22:1) within HT-29 and CaCo-2 cells were 0.15 and 0.37, respectively (Table 3).

### Effects of Nanoparticles on Apoptosis and Cell Cycle Distribution *in vitro*

**Figure 6:**
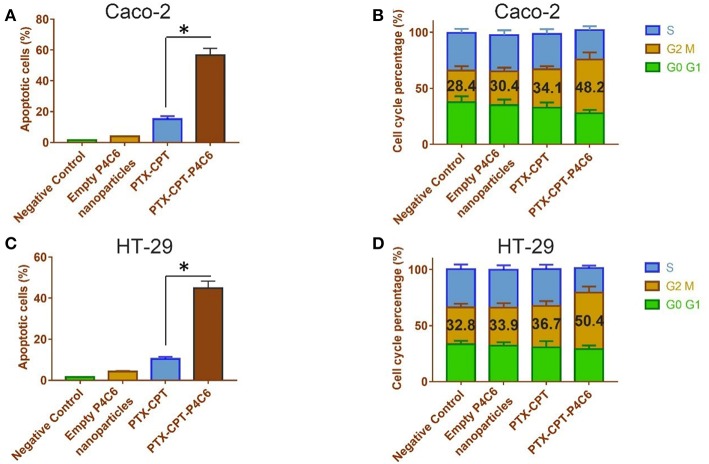
Effects of empty P4C6 nanoparticles, PTX-CPT and PTX-CPT-P4C6 on **(A,C)** apoptosis and **(B,D)** cell cycle distribution in Caco-2 and HT-29 cultures. Negative control cultures were left untreated. ^*^p < 0.05 (Student's t-test).

The induction of apoptosis by PTX-CPT-P4C6 was confirmed in flow cytometryic analysis (Figures 6A,C), where proportions of apoptotic Caco-2 and HT-29 cells were much higher after treatment with 0.3 μM PTX-CPT-P4C6 nanoparticles (44.9 ± 3.44 and 56.6 ± 4.5%) than after treatment with 0.3 μM PTX-CPT (10.4 ± 1.05 and 15.1 ± 1.9%). In other words, loading the two drugs into P4C6 nanoparticles increased their pro-apoptotic efficacy about 4-fold in Caco-2 cells and HT-29 cells.

Treating Caco-2 cells with 0.3 μM PTX-CPT-P4C6 for 48 h led to arrest of 48.2 ± 6.24% of cells in the G2/M-phase, compared to 30.4 ± 3.27 or 34.1 ± 2.79% of cells after treatment with empty P4C6 nanoparticles or the same concentration of PTX-CPT (*p* < 0.05; Figure 6B). Similarly, treating HT-29 cells with 0.3 μM PTX-CPT-P4C6 led to arrest of 50.4 ± 5.51% of cells in G2/M-phase, compared to 33.9 ± 4.08 or 36.7 ± 3.53% with free P4C6 nanoparticles or PTX-CPT (*p* < 0.05; Figure 6D).

These two sets of experiments suggest that apoptosis induction and mitotic arrest help explain the observed ability of micelle-encapsulated PTX and CPT to inhibit tumor cell proliferation.

### Effects of Nanoparticles on Invasion and Migration of HT-29 Cells *in vitro*

**Figure 7:**
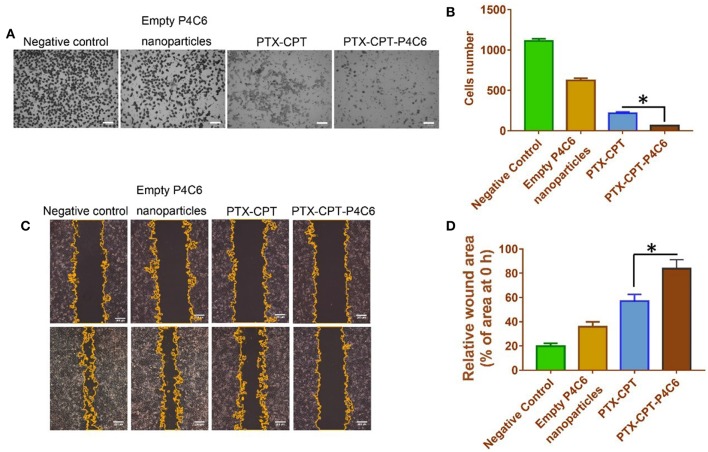
Effects of empty P4C6 nanoparticles, PTX-CPT and PTX-CPT-P4C6 on invasion and migration of HT-29 cells. **(A)** HT-29 cells were treated with different formulations in serum-free medium for 24 h in Boyden transwell chambers. Cells that reached the bottom of membranes were counted. Negative control cultures were left untreated. Scale bar, 100 μm. **(B)** Quantitation of the transwell assay. Each bar represents the mean ± SD of three independent observations. **(C)** Cells were scratched with a pipette tip, washed twice in PBS and photographed at 0 h, then treated with empty P4C6 nanoparticles, the mixture PTX-CPT, or PTX-CPT-P4C6. Negative control cells were left untreated. The experiment was allowed to proceed until the gap was nearly covered by migrated cells in negative control cultures (96 h). At the end of the experiment, migration was photographed under a phase-contrast microscope, and the gap area was measured using ImageJ software. Scale bar, 200 μm. **(D)** Quantitation of the wound healing assay. Each bar represents the mean ± SD of three independent measurements. ^*^p < 0.05 (Student's *t*-test).

To observe the effects of different formulations on the invasion of HT-29 cells in a three-dimensional setting, the Boyden transwell chamber assay was performed. The number of invasive cells in the PTX-CPT-P4C6 group (64 ± 14 cells per field of view) was significantly lower than that in the PTX-CPT group (219 ± 34; Figures 7A,B). Similarly, in the wound healing assay, the wounded area in the PTX-CPT-P4C6 group was 83.9 ± 7.2% of the original area after 96 h, compared to 57.1 ± 5.4% in the PTX-CPT group and 35.7 ± 4.2% in the empty P4C6 nanoparticles group (Figures 7C,D).

### Effect of Nanoparticles on HT-29 Human Colon Cancer Xenografts *in vivo*

**Figure 8:**
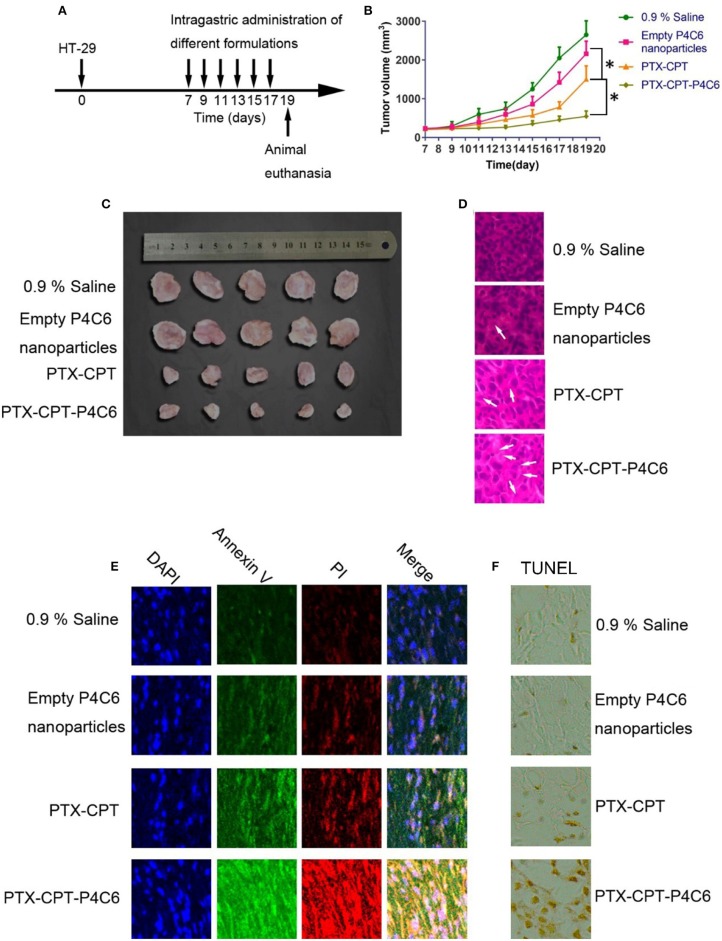
Anti-tumor effects of different formulations *in vivo*. **(A)** Schematic diagram showing the HT-29 xenograft model and the timing of drug administration. **(B)** Tumor growth during treatment with empty P4C6 nanoparticles, PTX-CPT and PTX-CPT-P4C6. Negative control cells were treated with 0.9% saline. **(C)** Photographs of excised HT-29 tumors removed on day 19. **(D)** Hematoxylin-eosin staining of tumor tissues removed on day 19. Magnification, ×400. Arrows indicate shrunken or fragmented nuclei. **(E)** Staining of tumor tissue on day 19 to detect early apoptosis (annexin V) and late apoptosis (propidium iodide, PI). **(F)** Immunohistochemical analysis of the degree of apoptosis in tumor tissues by TUNEL staining. Magnification, ×400. All results are from three independent experiments. Values are mean ± SD. ^*^p < 0.05 (Student's *t*-test).

The *in vivo* antitumor efficacy of the PTX-CPT-P4C6 was investigated on HT-29 human breast tumor-bearing nude mice. Animals were treated by oral gavage with PBS or different drug formulations every 2 days, when tumor volume were measured (Figures 8A,B). Tumor growth was significantly suppressed by PTX-CPT and PTX-CPT-P4C6, compared to 0.9% saline and empty P4C6 nanoparticles (Figures 8B,C). Hematoxylin-eosin staining of tumor tissue revealed more extensive tumor cell necrosis and larger numbers of shrunken and fragmented nuclei in PTX-CPT-P4C6 tumors than in other tumors (Figure 8D). PTX-CPT-P4C6 nanoparticles caused the greatest tumor inhibition, and tumors from these animals showed the greatest levels of early apoptosis (based on annexin V staining) and late apoptosis (based on PI staining; Figure 8E). Coincided with annexin V-PI assay, results of TUNEL staining confirmed PTX-CPT-P4C6 could cause highest level of apoptosis of HT-29 colon cancer cells among all treatment groups (Figure 8F).

### Preliminary Evaluation of Nanoparticle Toxicity

**Figure 9:**
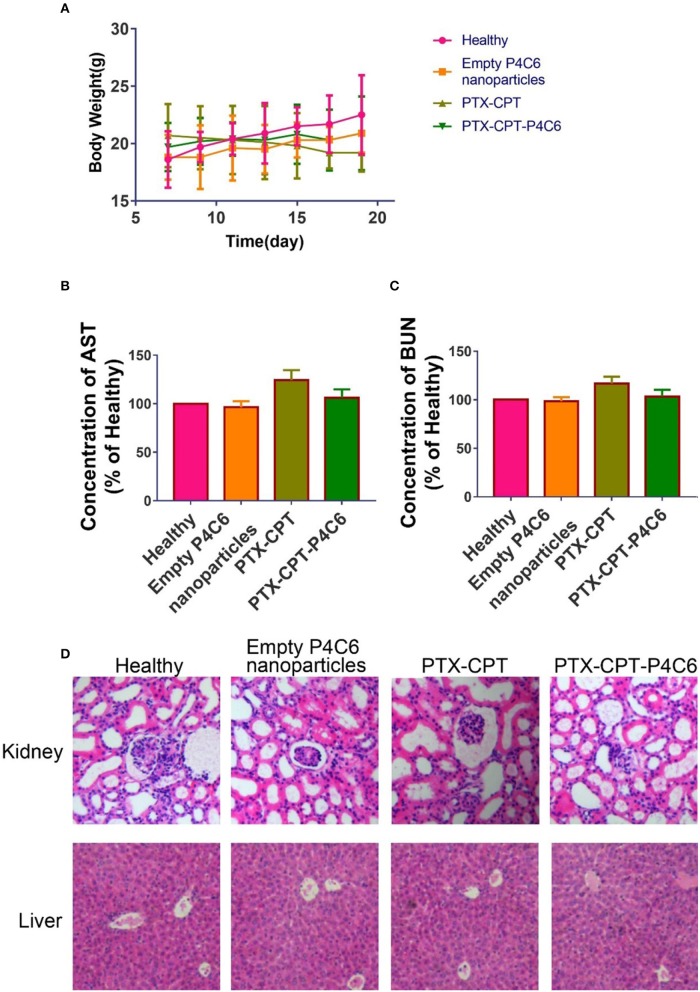
Preliminary toxicity evaluation of PTX-CPT-P4C6 *in vivo*. Assays were performed after 12 d of treatment. **(A)** Body weight during treatments. **(B,C)** Levels of aspartate aminotransferase (AST) and blood urea nitrogen (BUN). **(D)** Hematoxylin-eosin staining of kidney and liver sections. Magnification, ×200.

Mice were treated by oral gavage with PBS or different drug formulations every 2 days, when body weight was also measured (Figure 9A). No deaths or significant loss of body weight relative to healthy controls occurred with empty P4C6 nanoparticles, PTX-CPT, or PTX-CPT-P4C6. Aspartate aminotransferase and blood urea nitrogen levels after all treatments were similar to those in controls (Figures 9B,C), as was morphology of kidney and liver tissue based on hematoxylin-eosin staining (Figure 9D). No significant glomerular damage was observed in kidney sections, and broken hepatic cords were not seen in liver tissue. Negligible leukocyte migration to kidney or liver was observed.

## Discussion

Functional modification of calixarenes at the upper and/or lower rims makes it possible to derive a variety of molecules with tunable physicochemical properties and selectivity for guest molecules (Arena et al., 2000). They are also attractive for drug delivery because they can increase the poor solubility of many anticancer drugs, prolong the circulation of drugs by protecting them from premature interaction with host molecules, and improve drug penetration into the target tissue (Ngandeu Neubi et al., 2018). The present study focused on a phosphonated calix[4]arene (P4C6) capable of co-loading two commonly used anticancer agents, CPT and PTX: the CPT occupies an external bowl-shaped cavity, while PTX is solubilized between the bilayers of liposome.

The size, size distribution and zeta potential of nanoparticles are critical determinants of their toxicity (Shah and Dobrovolskaia, 2018) and bio-distribution (Graham et al., 2017). The average hydrodynamic diameters of all nanoparticles in this study can avoid renal excretion and evade detection by the mononuclear phagocytic system (Devarajan et al., 2010). All formulations showed low polydispersity, suggesting mono-disperse populations and narrow size distribution. Nanoparticles remained stable for at least 72 h over a broad range of pH values and multiple times dilution. These results suggest that these drug-loaded nanoparticles can remain intact until reaching the colon.

We formulated PTX-CPT-P4C6 nanoparticles with a CPT:PTX mass ratio of 8.79:3.88% per 1 g of nanoparticle, which corresponds to a molar ratio of 5.22:1. Screening a few ratios of CPT:PTX showed 5.22:1 to be most effective in cytotoxicity assays *in vitro*. We performed these assays using Caco-2 cells, which are heterogeneous human epithelial colorectal adenocarcinoma cells (Schreck and Melzig, 2018) used most often as a confluent monolayer rather than individual cells (Ellens et al., 2018); the monolayer can, under certain conditions, form a polarized epithelial cell monolayer (Gibaud and Attivi, 2012). Therefore Caco-2 was used here to mimic normal colorectal epithelial cells (Beloqui et al., 2016). The most common form of colon cancer is adenocarcinoma (Beloqui et al., 2016; Yueh et al., 2016), so we selected HT-29 adenocarcinoma cells as a colon cancer model. These cells are frequently used in tumorigenicity studies (Al-Saffar et al., 2018; Guerrero et al., 2018; Liu et al., 2018) and can form well-differentiated adenocarcinoma consistent with primary colon cancer in nude mice (Handali et al., 2018). We found PTX-CPT-P4C6 nanovesicles to suppress proliferation of HT-29 cells more than Caco-2 cells. The reasons and implications of this require further investigation.

Calixarenes are formed by the reaction of para-substituted phenol with formaldehyde. Calixarenes are aromatic macrocyclic compounds, which can form host-guest complexes with different small molecules after macrocyclic modification (Mo et al., 2015). The host-guest complexes formed between various calixarene derivatives and quaternary ammonium compound (such as trimethyllysine) have been well studied (Georghiou et al., 2018; Buldenko et al., 2019; Xu et al., 2019). Daze et al. found that p-sulfonated calix[4]arenes can specifically bind trimethylated lysine of different amino acids, non-methylated lysine and trimethylated lysine (Daze et al., 2012). The binding of cationic proteins or polypeptides with these host macrocyclic molecules is quite common (Adhikari et al., 2014; Cinà et al., 2017). Allen's group found that the affinity of p-sulfonated calix[4]arenes with H3K9Me3 was significantly higher than that of H3K9Me0 (Allen et al., 2014).

Our lab has been engaging in research on the use of phosphonated calix[4]arenes as carrier to improve the anti-cancer efficacy. In previous study, we found that P4C6 had a suitable size of cavity to accommodate cationic drug carboplatin to form host-guest complex. Then the amphiphilic phosphonated calix[4]arenes were obtained by properly modifying the lower rim of phosphonated calix[4]arenes with n-alkyl groups. The resultant drug-loaded vesicles can improve carboplatin accumulation within the tumor site. In another project, we loaded curcumin by P4C6 to form a core-shell structure micelle. Compared with free curcumin, the drug-loaded micelles can significantly reduce the number of CD44^+^/CD133^+^ cancer stem cells in triple-negative breast cancer mice model. The mechanism was that drug-loaded micelles can reduce the expression of β-catenin in the nucleus. In the third study, we found, compared to free drugs, drugs loaded P4C6 showed stronger apoptosis induction as well as invasion and self-renewal capacity suppression in human ovarian cancer SKOV-3 cells. It was further found that the molecular mechanism of the above drugs-loaded nanoparticles may be through preventing JMJD3 (epigenetic regulator) binding with H3K27me3 (suppressor of transcription), thereby protecting the lysine trimethylation of H3K27me3 and antagonizing the effect of JMJD3, finally promoting the differentiation of ovarian cancer stem cells by reducing the transcription of the oncogene HER2. Through the above researches, we found that P4C6 loaded with different anticancer drugs can produce synergistic effect. P4C6 seems to be able to downregulated JMJD3 expression to modulate the H3K27me3 epigenetic mark of the promoters of HER2 and MYCN.

## Conclusion

The present study describes a new phosphonic acid calixarene derivative consisting of a single amphiphilic compound, P4C6, which can encapsulate CPT in the calixarene cavity and PTX among the hexane tails. The optimized PTX-CPT-P4C6 nano-formulation shows efficient drug loading, small size, and low polydispersity. The nanoparticles were taken up efficiently by two colon cancer cell lines and showed greater cytotoxicity than a simple mixture of the two drugs. This cytotoxicity was associated with apoptosis induction, cell cycle arrest and suppression of invasion and migration. PTX-CPT-P4C6 inhibited HT-29 adenocarcinoma cells, which represent the most common form of colon cancer. PTX-CPT-P4C6 showed the greatest inhibition of HT-29 tumors in mice, with negligible side effects. Therefore, the PTX-CPT-P4C6 nano-formulation holds promise for improving the efficacy of PTX and CPT combination therapy against colon cancer.

## Data Availability Statement

The raw data supporting the conclusions of this manuscript will be made available by the authors, without undue reservation, to any qualified researcher.

## Ethics Statement

The animal study was reviewed and approved by approved by the Ethics Committee of Guilin Medical University (Ethics number YXLL-2016-088).

## Author Contributions

MeL carried out biological experiments. MC and MiL synthesized and characterized nanomaterials. KW performed drug loading and releasing trials. LM analyzed data and review the manuscript. JM wrote the paper and led the research.

### Conflict of Interest

The authors declare that the research was conducted in the absence of any commercial or financial relationships that could be construed as a potential conflict of interest.
