# Supplementary material for: Characterization of an Amphiphilic Phosphonated Calixarene Carrier Loaded With Carboplatin and Paclitaxel: A Preliminary Study to Treat Colon Cancer in vitro and in vivo
Source: Front Bioeng Biotechnol. 2019 Oct 1;7:238. doi: 10.3389/fbioe.2019.00238 (PMC6779836; doi:10.3389/fbioe.2019.00238)
Supplement: Supplementary file 1 [file Table_1.DOCX]

**Characterization of an amphiphilic phosphonated calixarene carrier loaded with carboplatin and paclitaxel: a preliminary study to treat colon cancer *in vitro* and *in vivo***

Meiying Li^1,2#^, Liujun Mao^4#^, Meirong Chen^3^, Mingxin Li^2^, Kaixuan Wang^2^, Jingxin Mo^1^*

^1^Clinical Research Center for Neurological Diseases of Guangxi Province, The Affiliated Hospital of Guilin Medical University, Guilin 541001, China,

^2^School of Pharmacy, Guilin Medical University, Guilin 541001, China,

^3^Department of Graduate, The Affiliated Hospital of Guilin Medical University, Guilin 541001, China

^4^Department of Further-education, The Affiliated Hospital of Guilin Medical University, Guilin 541001, China.

**Quantification assay by LC/TOF MS**

Liquid chromatography–time of flight–mass spectrometry (LC/TOF MS) was used for the simultaneous quantification of CPT and PTX in drugs loaded nanoparticles. The separation of CPT and PTX on a Waters HPLC SunFire C18 column at a flow rate of 0.25 ml/ min using gradient elution mode. The total analytical time was 12 min. Detection and quantitation was performed by electrospray ionization (ESI) in the positive ionization mode with selective ion monitoring (SIM) at m/z 310.0152 for CPT and 876.3224 for PTX. The calibration curves were linear over the concentration range of 10–4,000 ng/ml for CPT and 5–2,000 ng/ml for PTX (r^2^>0.99), with the respective lower limit of quantification (LLOQ) at 10 and 5 ng/ml.


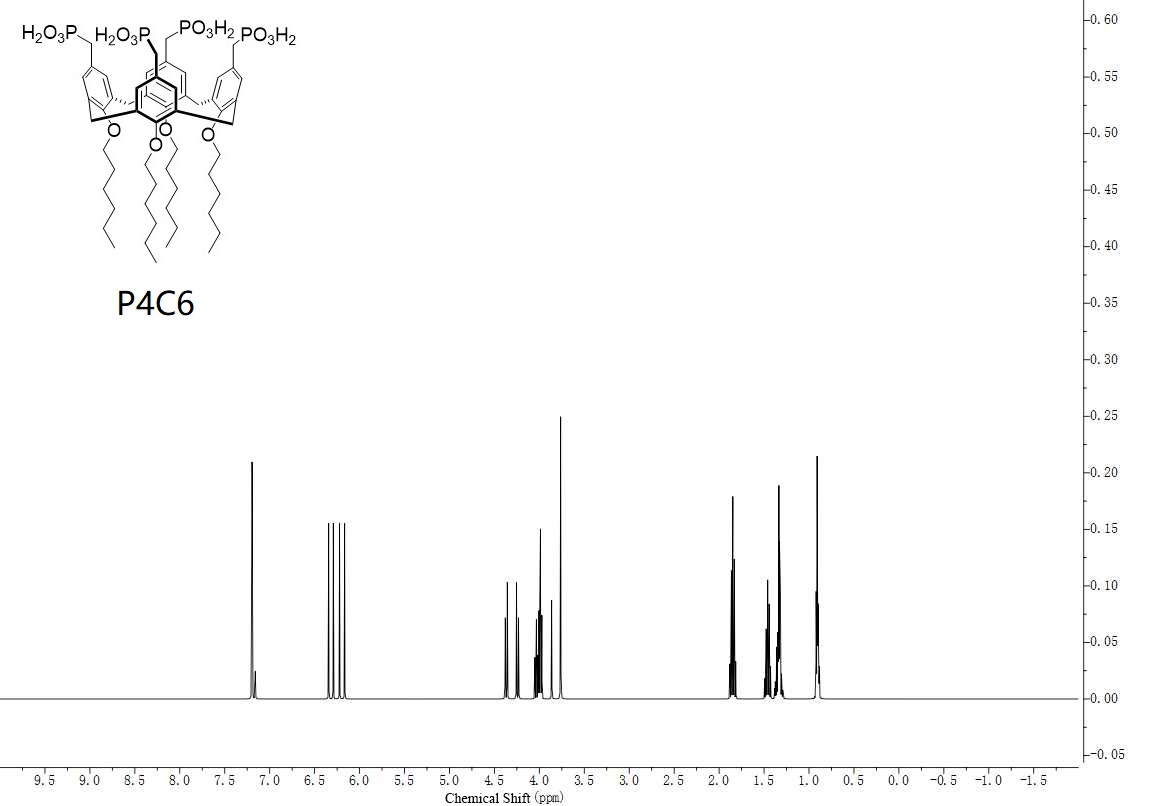


**Figure S1.** ^1^H NMR spectrum of P4C6.


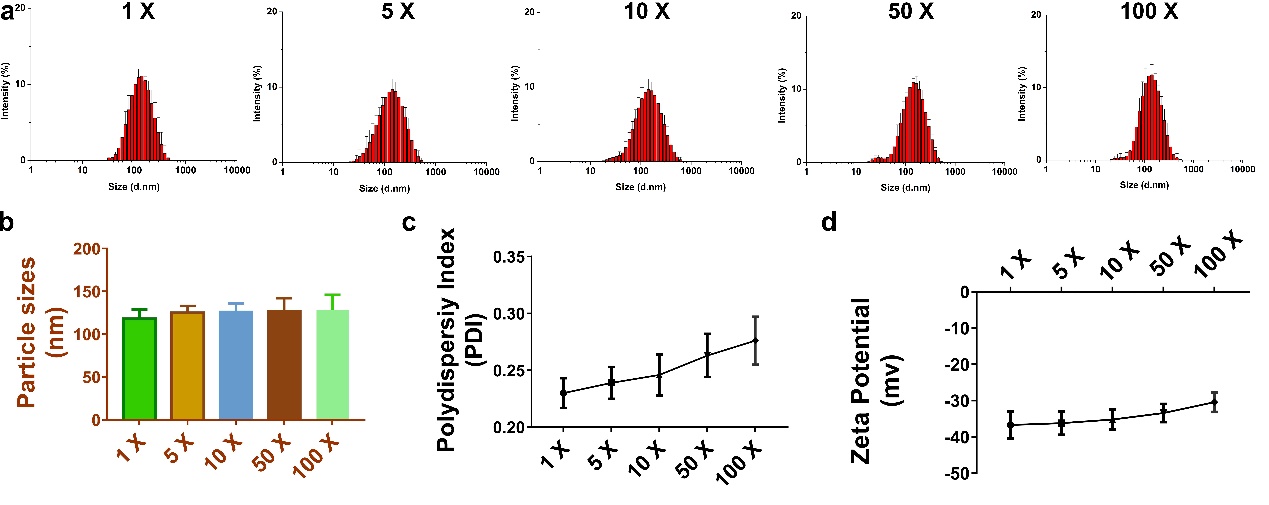


**Figure S2.** Changes of (a) particle size distributions, (b) mean particle sizes, (c) polydispersity index and (d) zeta potentials of 0.3 μM PTX-CPT-P4C6 dispersed in 0.9 % saline for up to 100 times. Error bars indicate SD (n = 3).


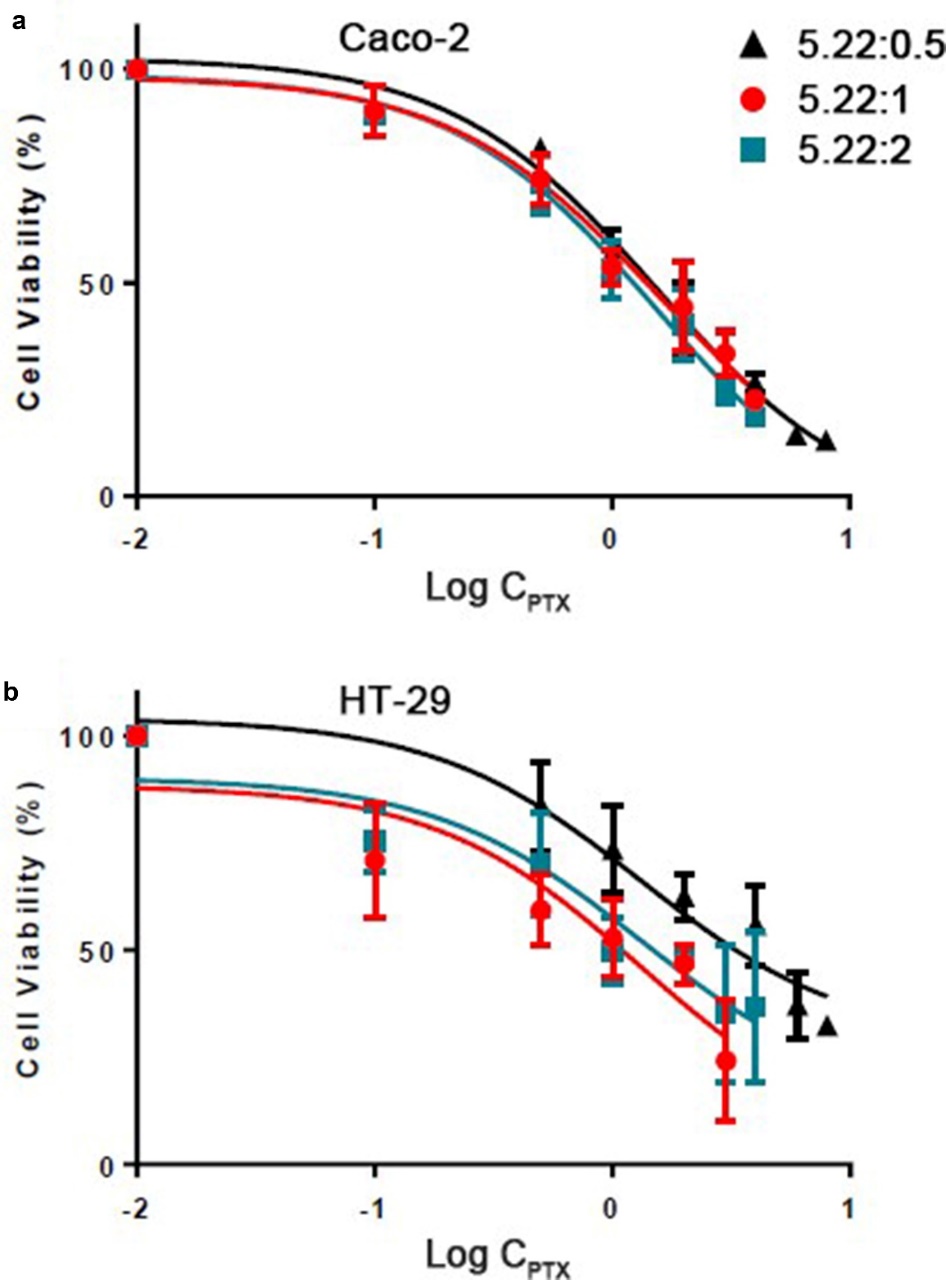


**Figure S3.** Viability of (a) Caco-2 cells and (b) HT-29 cells after 24-h treatment with increasing concentrations of a simple mixture of CPT and PTX in the indicated molar ratios. Viability was measured using the MTT assay. In both the mixture and nanoparticles, the molar ratio CPT:PTX was 5.22:1.


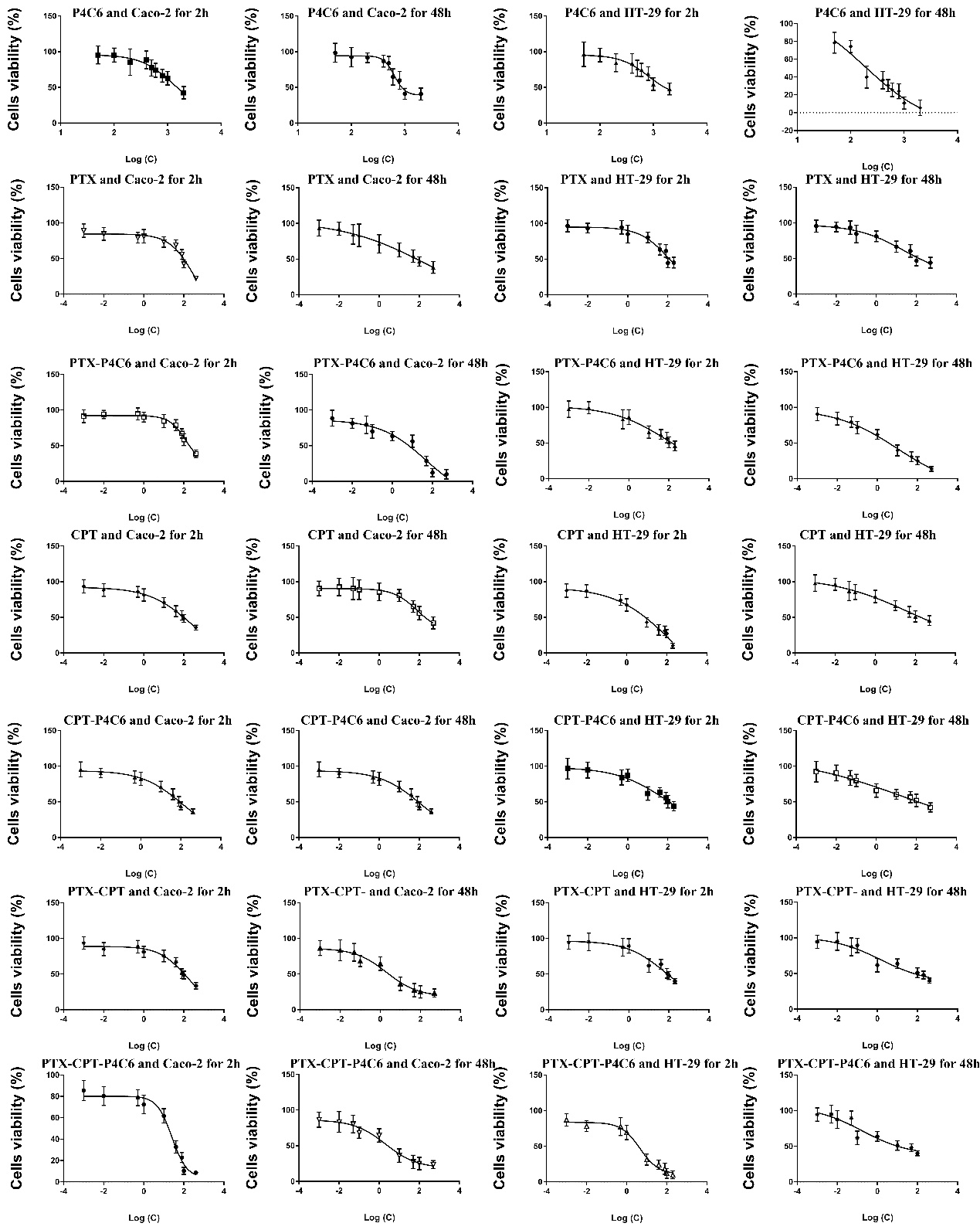


**Figure S4.** Dose-response curves comparing the IC_50_ of empty P4C6 nanoparticle, a simple mixture of PTX and/or CPT, and PTX- and/or CPT-P4C6 against HT-29 and Caco-2 cells. In all formulations, the molar ratio CPT:PTX was 5.22:1.
